# Thymic DCs derived IL-27 regulates the final maturation of CD4^+^ SP thymocytes

**DOI:** 10.1038/srep30448

**Published:** 2016-07-29

**Authors:** Hui Tang, Jie Zhang, Xiuyuan Sun, Xiaoping Qian, Yu Zhang, Rong Jin

**Affiliations:** 1 Key Laboratory of Medical Immunology, Department of Immunology, Ministry of Health, School of Basic Medical Sciences, Peking University Health Science Center, 38 Xue Yuan Road, Beijing, China

## Abstract

IL-27, as a pleiotropic cytokine, promotes the differentiation of naïve T cells to Th1, while suppressing Th2 and Th17 differentiation in the periphery. However, the role of IL-27 in the thymocyte development remains unknown. Here we showed that IL-27 was highly expressed in thymic plasmacytoid dendritic cells (pDCs) while its receptor expression was mainly detected in CD4^+^ single-positive (SP) thymocytes. Deletion of the p28 subunit in DCs resulted in a reduction of the most mature Qa-2^+^ subsets of CD4^+^ SP T cells. This defect was rescued by intrathymic administration of exogenous IL-27. *In vitro* differentiation assay further demonstrated that IL-27 alone was able to drive the maturation of the newly generated 6C10^+^CD69^+^CD4^+^ SP cells into Qa-2^+^ cells. Collectively, this study has revealed an important role of thymic DCs-derived IL-27 in the regulation of the phenotypic maturation of CD4^+^ SP thymocytes.

Thymus is the main site of T lymphopoiesis. On the basis of CD4 and CD8 expression, the process of thymocyte development can be divided into three major stages, double-negative (DN), double-positive (DP) and single-positive (SP)^1^,^2^,^3^. Both CD4 SP and CD8 SP thymocytes exhibit significant heterogeneity in phenotype, featured by the expression of 6C10, CD69 and heat-stable antigen (HSA) at the early stage and the acquisition of Qa-2 at the late stage^4^,^5^,^6^,^7^,^8^. Using these surface markers, we have defined a four-stage developmental program for CD4 SP thymocytes and demonstrated that the progression from SP1 to SP4 is coupled with a steady increase in the proliferative response and cytokine secretion^9^,^10^.

The maturation of SP thymocytes is critically dependent on the structural and functional integrity of the thymic medulla. The progression from SP3 to SP4, for example, was shown to be severely blocked in Relb- and Aire-deficient mice^9^,^11^. Deletion of the transcriptional repressor NKAP, which binds to HDAC3, impaired the final maturation of CD4 SP thymocytes in addition to an early developmental defect at the transition from DN3 to DP stages^12^. Nevertheless, the exact signal driving single positive thymocyte maturation remains enigmatic.

IL-27 is composed of two subunits, p28 (also named IL-27p28, or IL-30) and EBV induced gene 3 (EBI3)^13^,^14^. It signals through a heterodimeric receptor gp130 and IL-27RA (also known as TCCR and WSX-1)^15^,^16^. Multiple biological activities have been reported for IL-27^17^,^18^. In the immune system, IL-27 receptor expression is detected on a wide array of cells, including antigen presenting cells (such as monocytes, DCs and macrophages) and T and B lymphocytes^19^,^20^,^21^,^22^,^23^. During the early stage of immune responses, it has been described as a Th1-polarizing mediator and inflammation promoter by acting directly on naive T cells and sensitizing CD4^+^ T cells to respond to IL-12 stimulation, through early induction of the transcription factor T-bet^13^,^24^. In the late phase of infection, however, IL-27 appears to have an anti-inflammatory role, suppressing the development of Th1, Th2 and Th17 subsets^19^,^25^,^26^,^27^. A recent study reported that CD4^+^ T cells from CD11c-p28^flox/flox^ conditional knockout mice, upon TCR or mitogen stimulation, showed higher levels of IFN-γ production and T-bet expression^28^. However, the role of IL-27 in the thymocyte development remains poorly defined.

In the present study, we showed that pDCs was the main source of IL-27 in the thymus. DC-specific deletion of the gene encoding IL-27p28 resulted in the reduction of Qa-2^+^ CD4 SP T cells in the thymus, supporting that it is critically involved in the phenotypic maturation of CD4^+^ SP thymocytes.

## Materials and Methods

### Mice

All the animal procedures were conformed to the Chinese Council on Animal Care Guidelines and were approved by the ethics committee of Peking University Health Science Center with an approval number of LA2014178. C57BL/6J (B6) mice were purchased from the Department of Laboratory Animal Science of Peking University Health Science Center (Beijing, China). CD11c-cre p28^flox/flox^ mice on a C57BL/6 background were kindly provided by Dr. Zhinan Yin from Nankai University (Tianjin, China). All mice were kept in specific pathogen-free condition. Age-matched (6–8 weeks) female mice were used in all experiments.

### Reagents

Anti-CD8 (3.155) was prepared from a hybridoma obtained from American Type Culture Collection (Manassas, VA). The FACS antibody 6C10 (SM6C10) was kindly provided by Dr. Linna Ding (National Institutes of Health) and was subsequently labeled with FITC. Alexa Fluor@ 647-conjugated anti-mouse Qa-2 was purchased from BioLegend (San Diego, CA). All other FACS antibodies used in the study were purchased from BD PharMingen (San Diego, CA). Recombinant mouse IL-27 and IL-27 neutralizing antibody (Anti-IL-27p28) were purchased from R&D Systems (Minneapolis, MN). Anti-STAT1, anti-phospho-STAT1, anti-STAT3, anti-phospho-STAT3 and anti-β-actin used in western blot were purchased from R&D Systems (Minneapolis, MN).

### Flow cytometric analysis

Single cell suspensions were prepared from the thymus of CD11c-cre p28^flox/flox^ mice and WT littermates. Cells were then stained with mAbs against CD4, CD8, CD25, CD44, NK1.1, 6C10, CD69 and Qa-2 for 20 min at 4 °C. FACS analysis was conducted on BD FACS Calibur or Beckman Coulter Gallios. Data were analyzed with Flowjo Software (Treestar, USA) or Kaluza Flow Cytometry Analysis (Beckman Coulter). Intracellular staining of IL-27 was performed as described^10^.

### Thymic stromal cell isolation

The thymus was cut into small pieces (<1 mm^3^). Tissue fragments were digested with 2 mg/ml collagenase type IV and 0.01 mg/ml DNaseI at 37 °C for about 1–2 hours with constant shaking. After removal of undigested fragments, the stromal cells were enriched by density gradient centrifugation. In some experiments, contaminant thymocytes were further deleted by immuno-magnetic bead depletion. To purify thymic DCs, cells were stained with fluorescence-labeled antibodies against CD11c, CD45RA, CD8α and CD172α and sorted on a BD FACS Aria II into the following fractions: total DCs (CD11c^+^), pDCs (CD11c^int/hi^CD45RA^+^), resident conventional DCs (CD11c^+^CD8α^+^CD172α^−^), and migratory DCs (CD11c^+^CD8α^−^CD172α^−^). The purity of the sorted DC was 95%. To isolate macrophages and thymic epithelial cells (TECs), stromal cells were stained with mAbs against CD11b, F4/80, CD45, Epcam and Ly51. Macrophages were identified as CD11b^+^F4/80^+^, cTECs as CD45^-^Epcam^hi^Ly51^+^ and mTECs as CD45^−^Epcam^hi^Ly51^−^.

### Purification of thymocyte subsets and naïve T cells

Single-cell suspensions of total thymocytes were stained for 20 min at 4 °C in FACS buffer (BSS with 2%FCS) with CD4, CD8 mAbs. Cell sorting was performed to purify DN (CD4^−^CD8^−^), DP (CD4^+^CD8^+^), CD4^+^ SP (CD4^+^CD8^−^) and CD8^+^ SP (CD4^−^CD8^+^) T cells. To purify CD4 SP subsets, total thymocytes were first treated with anti-CD8 (3.155) mAb and complement (guinea pig sera) to deplete CD8^+^cells. After removal of dead cells by density centrifugation, the viable cells were stained for CD4, CD8, CD25, NK1.1, 6C10, CD69, Qa-2. SP1 (6C10^+^CD69^+^), SP2 (6C10^−^CD69^+^), SP3 (CD69^−^Qa-2^−^) and SP4 (CD69^−^Qa-2^+^) cells were sorted by gating on CD4^+^CD8^−^CD25^-^NK1.1^−^ cells.

For the isolation of CD4^+^ naive T cells, cells from lymph nodes (including axillary, mesenteric, and inguinal lymph nodes) were stained for CD4, CD8, CD44 and CD62L. Sorting was performed to obtain CD4^+^CD8^−^CD44^lo^CD62L^hi^ cells.

### Polymerase chain reaction (PCR)

Genomic DNA was extracted using Mouse Tail Direct PCR Kit (Foregene). Genotype was determined by PCR using the following primer sets: p28-flox, forward TCCCTTCCAGGACCATACTGCTAA, reverse ACCCAAACACAGGCCAGTACTCTA; cre, forward GCCTGCATTACCGGTCGATGC, reverse GCCTGCATTACCGGTCGATGC.

RNA was isolated from freshly isolated cells or cells cultured under different conditions using Trizol reagent. cDNA was synthesized using reverse transcription kit (Progema). For quantitative PCR, iQ SYBR Green Supermix (Bio-Rad Laboratories) was used according to the manufacturer’s instructions. The amplification was performed on an iCycler (Bio-Rad Laboratories). GAPDH was used as an internal control. The primers used in the study were listed in supplementary Table 1.

### *In vitro* cell culture

1 × 10^6^ SP1 cells were cultured in RPMI1640 with 10%FCS in 24-well plates in the presence or absence of 1 × 10^5^ thymic DCs. IL-27 (2 ng/ml), IL-30 (IL-27p28) (10 ng/ml) and/or anti-IL-27p28 (1 μg/ml) were included in the culture if necessary. At various time points, the culture was harvested and analyzed for the generation of Qa-2^+^ cells by flow cytometry.

### IL-27 intrathymic injection

Intrathymic injection was performed as described^10^. In brief, 20 μl IL-27 (8 μg/ml) was injected intrathymically using a Hamilton syringe. The control mice were injected with 20 μl PBS. After 48 hours post injection, the recipient mice were sacrificed and analyzed for thymocyte development.

### Western Blotting

Freshly isolated CD4 SP thymocytes from CD11c-cre p28^flox/flox^ mice and littermates were lysed in lysis buffer (20 mM Tris pH 8.0, 137 mM NaCl, 5 mM EDTA, 10% glycerol, 1% Triton X-100, 1 mM PMSF, 1 mM aprotinin, 1 mM leupeptin, 1 mM EGTA, 1 mM Na3VO4, 1 mM tetrasodium pyrophosphate, and 10 mM NaF). The lysate was resolved on a 12% reducing SDS-polyacrylamide gels and transferred to a polyvinylidene difluoride membrane. After blocking with Tris-buffered saline (pH 7.4) containing 5% dried skimmed milk, the membrane was incubated with anti-STAT1. anti-phospho-STAT1, anti-STAT3. anti-phospho-STAT3, or anti-β-actin (R&D Systems, Minneapolis, MN).), followed by probing with HRP-conjugated anti-rabbit antibody (Sigma Aldrich). The immunoreactive bands were detected by chemiluminescence with ECL detection reagents (Life Technologies, Grand Island, NY, USA).

### Statistics

Data are presented as mean ± SD from three or more independent experiments. Statistical analysis was performed using a two way ANOVA t test. A *p* value < 0.05 was considered statistically significant.

## Results

### The thymic expression of IL-27 and its receptors

**Figure 1:**
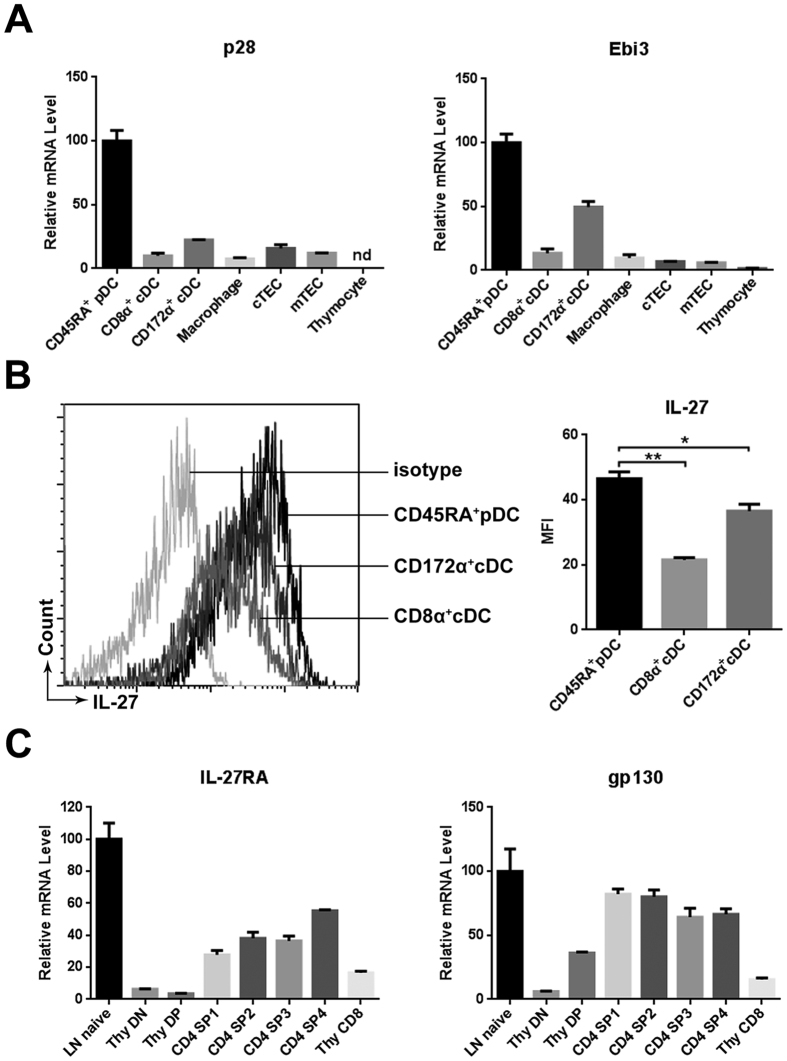
IL-27 and its receptor expression in the thymus. (**A**) Total thymocyte, CD11c^int^CD45RA^+^pDC, CD11c^hi^CD8α^+^CD172α^−^ and CD11c^hi^CD8α^−^CD172α^+^ cDC, CD45^−^Epcam^hi^ Ly51^+^ cTEC, CD45^-^Epcam^hi^ Ly51^-^ mTEC and CD11b^+^F4/80^+^ macrophage were isolated from C57BL/6J mice. The expression of p28 and EBI3 mRNA was evaluated by quantitative PCR. nd, no detectable. (**B**) IL-27 protein expression in pDC, CD8α^+^ and CD172α^+^ cDC was analyzed by flow cytometry following intracellular staining with anti-IL-27. Representative histograms are shown on the left and the mean fluorescence intensity (MFI) on the right. (**C**) Purified DN, DP, CD8 SP and the four subsets of CD4 SP (SP1-4) thymocytes were analyzed for IL-27RA and gp130 mRNA expression using quantitative PCR. The lymph node (LN) naïve T cell was used as a positive control. All the experiments were repeated for more than three times and the data are shown as mean ± s.d. *p < 0.05; **p < 0.01.

To investigate the potential role of IL-27 in thymocyte development, we first examined the expression of IL-27 and its receptors in various thymic cell populations. The thymic DCs are composed of three major subsets, including CD11c^int^CD45RA^+^ plamacytoid DC (pDC) CD11c^hi^CD8α^+^CD172α^−^ thymus-derived conventional DC (cDC) and CD11c^hi^CD8α^−^CD172α^+^ migratory cDC. DCs subsets, cTECs (CD45^-^Epcam^hi^Ly51^+^), mTECs (CD45^−^Epcam^hi^Ly51^−^) and macrophages (CD11b^+^F4/80^+^) were isolated by cell sorting and quantitative PCR was performed to measure mRNA expression of p28 and EBI3, the two composing subunits of IL-27. Among the thymic stromal cells, DCs, especially pDCs, showed the highest levels of p28 and EBI3 mRNA expression. In contrast, they were hardly detected in total thymocytes (Fig. 1A). We further examined IL-27 protein expression in thymic DCs through intracellular staining with anti-IL-27. Again, pDCs displayed the strongest staining (Fig. 1B).

Peripheral naïve T cells, NK and NKT cells are good responder of IL-27^13^,^29^. To identify the target cells of IL-27 in the thymus, we analyzed expression of IL-27RA and gp130 in thymocytes. Compared with DN, DP and CD8 SP cells, each subset of CD4 SP thymocytes had an increased expression of IL-27RA and gp130 mRNA, approaching approximately half of the level in naive T cells (Fig. 1C). Therefore, CD4 SP thymocytes may represent the major IL-27-responding population in the thymus.

### Reduction of CD69^−^Qa^−^2^+^ SP4 thymocytes in CD11c-cre p28^flox/flox^ mice

**Figure 2:**
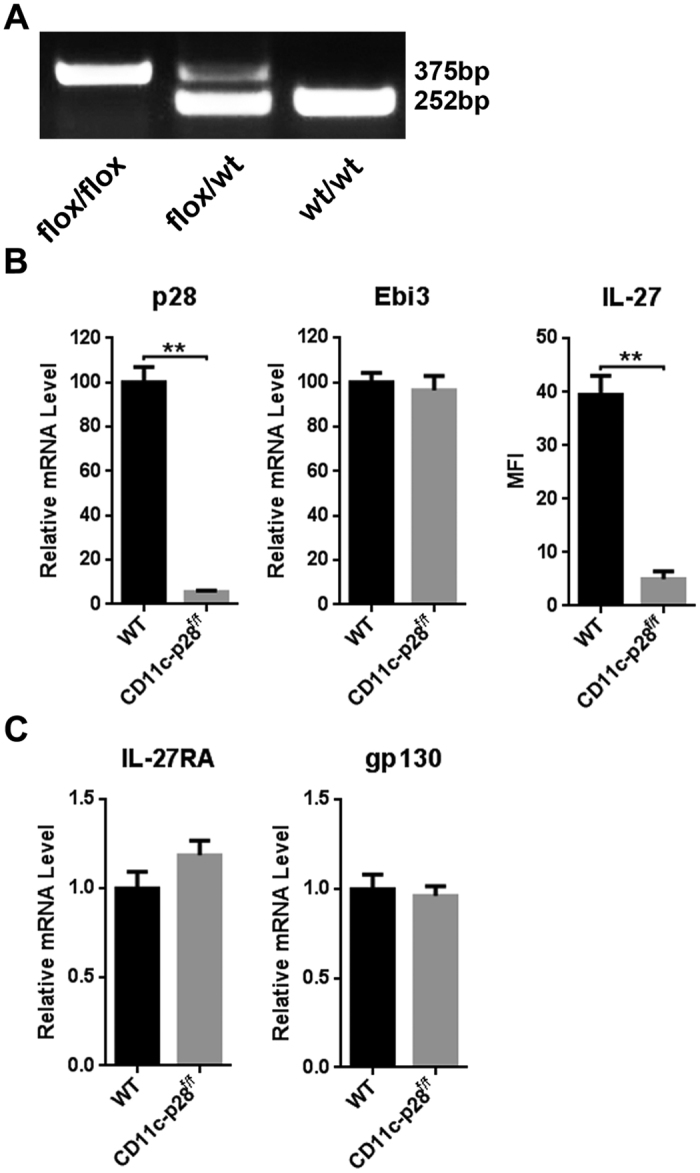
Deletion of p28-coding gene in DCs. (**A**) Mouse genotyping was performed using PCR. (**B**) p28 and Ebi3 mRNA levels were determined by quantitative PCR in total thymic DCs form CD11c-cre p28^flox/flox^ and WT mice. The IL-27 protein expression was measured by flow cytometry. (**C**) IL-27RA and gp130 mRNA expression was compared in CD4 SP thymocytes form CD11c-cre p28^flox/flox^ and WT mice. The experiments shown in B and C were repeated for more than three times and the data are presented as mean ± s.d. **p < 0.01.

Given the highly restricted expression of IL-27 in thymic DCs, we sought to determine the impact of DC-specific deletion of p28 on thymocyte development using CD11c-cre p28^flox/flox^ mice previously reported by Zhang *et al*.^28^. PCR analysis revealed the effective disruption of the gene encoding p28 in the genome (Fig. 2A), which was accompanied by a sharp reduction in p28 mRNA and IL-27 protein expression in purified thymic DCs (Fig. 2B). In contrast, the expression of EBI3, another composing subunit of IL-27, was not affected (Fig. 2B). In consideration of the possibility that ligand deficiency may alter the expression of cognate receptors, we also examined IL-27RA and gp130 expression in CD4 SP thymocytes. Comparable mRNA levels of IL-27RA and gp130 were detected in CD11c-cre p28^flox/flox^ mice and the WT littermates (Fig. 2C).

**Figure 3:**
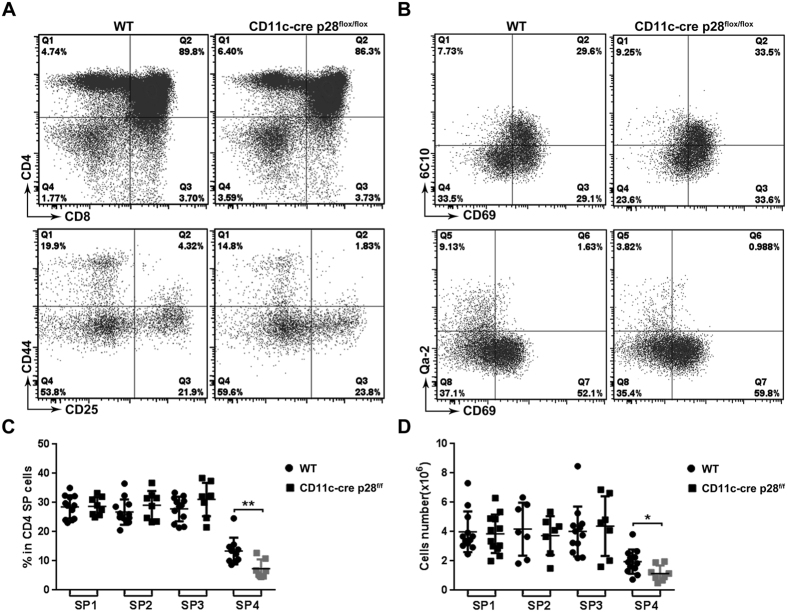
Defect in thymocyte development in CD11c-cre p28^flox/flox^ mice. Single cell suspension was prepared from the thymus of CD11c-cre p28^flox/flox^ mice and littermate controls. T cell development was analyzed by flow cytometry. (**A**) Representative dot plots showing CD4/CD8 staining of total thymocytes (upper) and CD44/CD25 staining of cells in the CD45^+^CD4^−^CD8^−^ gate (lower). (**B**) Representative dot plots showing 6C10/CD69 (upper) and CD69/Qa-2 (lower) staining of CD4^+^CD8^−^CD25^−^NK1.1^−^ thymocytes. (**C,D**) The percentage (**C**) and absolute cell number (**D**) of the four subsets of CD4 SP thymocytes subsets in CD11c-cre p28^flox/flox^ mice and littermate. Data were collected from 12 pairs of mice. Each dot or square represents an individual mouse and the horizontal bar denotes the mean. *p < 0.05; **p < 0.01.

Thymocyte development in the CD11c-cre p28^flox/flox^ mice was subsequently analyzed by flow cytometry. The KO and WT mice showed similar patterns of CD4/CD8 staining for total thymocytes and CD44/CD25 staining for DN thymocytes (Fig. 3A) and contained equal numbers of DN, DP, CD4 SP and CD8 SP cells (data not shown). In view of the high expression of IL-27R in CD4 SP thymocytes, a detailed analysis was performed for this population. We had previously defined a four-stage developmental program for CD4 SP thymocytes on the basis of cell surface expression of 6C10, CD69 and Qa-2. The SP4 stage, which was featured by Qa-2 expression, represents the most mature one^4^. Although there was no obvious difference in the total number of CD4 SP thymocytes, p28-deficiency resulted in a significant decrease of CD69^−^Qa-2^+^ SP4 cells (Fig. 3B–D), indicating that IL-27 was involved in the final maturation of CD4 SP thymocytes.

### Enhanced production of Qa-2^+^ cells by IL-27 in cultures of SP1 thymocytes

**Figure 4:**
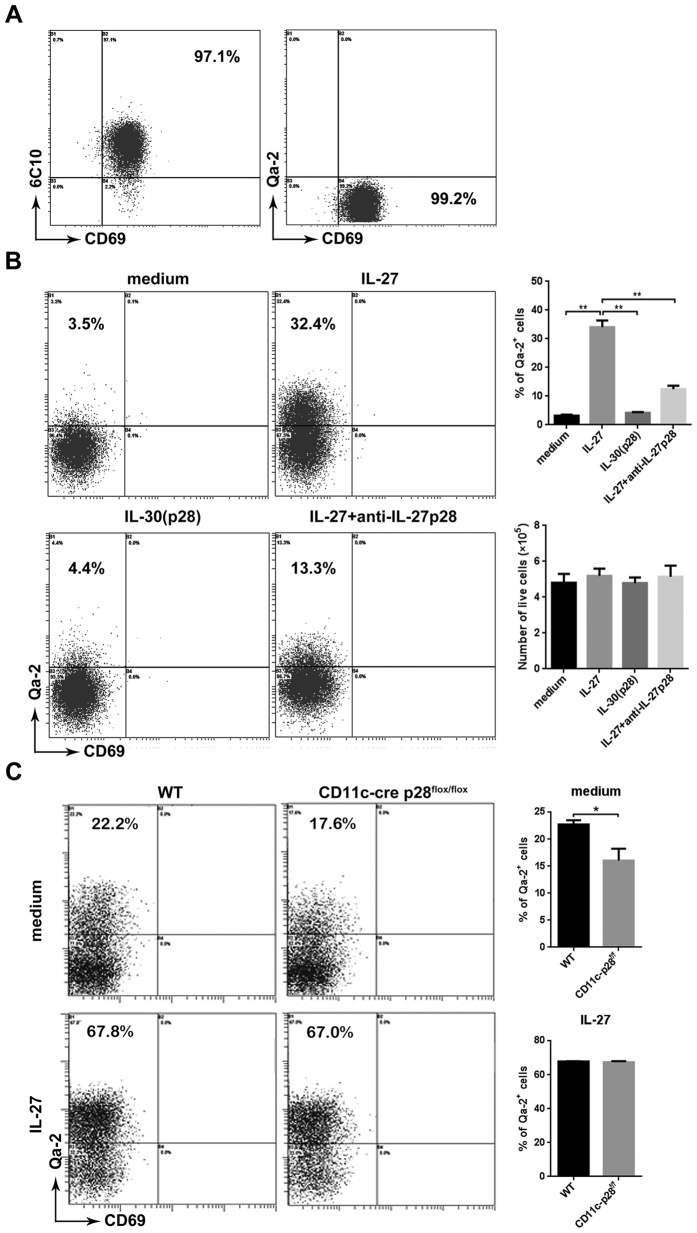
IL-27-enhanced development of Qa-2^+^ cells in CD4^+^ SP1 thymocyte cultures. (**A**) The profile of isolated CD4^+^ SP1 thymocytes was shown by the expression of 6C10 and CD69 (left), or by the expression of CD69 and Qa-2 (right). The purity of the sorted cells was 97–99%. (**B**) Freshly isolated CD4^+^ SP1 thymocytes from WT mice were cultured for 4 days in RPMI-1640 medium with or without the addition of IL-27, IL-30 or anti-IL-27p28. Acquisition of Qa-2 expression was detected by surface staining. Representative dot plots (left) and the percentage of Qa-2^+^ cells as well as the recovered live cells number (right) were presented. (**C**) SP1 cells were co-cultured with thymic DCs obtained from CD11c-cre p28^flox/flox^ or WT mice for 4 days in the presence or absence of IL-27. Qa-2 expression was evaluated by FACS analysis. Representative dot plots (left) and the percentage of Qa-2^+^ cells (right) are presented. The experiments were repeated for five times. *p < 0.05; **p < 0.01.

It has been previously shown that purified SP1 cells, when co-cultured with thymic DCs or TECs, were able to undergo phenotypic maturation, leading to the generation of Qa-2^+^ cells^9^,^30^. This system provided an opportunity to directly examine the impact of IL-27 on the maturation of CD4 SP thymcoytes *in vitro*. The profile and purity of freshly isolated CD4^+^ SP1 cells was shown in Fig. 4A. Notably, IL-27 alone induced a substantial number of Qa-2^+^ cells. Such an effect was abolished with the addition of anti-IL-27 (Fig. 4B), confirming the specificity of IL-27 action. Similar numbers of live cells were recovered from the cultures with or without IL-27 (Fig. 4B), ruling out the possibility that IL-27 preferentially expanded contaminating Qa-2^+^ cells in the culture. As the p28 subunit is reported to be able to act independently as IL-30, we also test the biological activity of IL-30 in similar cultures. As shown in Fig. 4B, IL-30 failed to support the production of Qa-2^+^ cells.

Thymic DCs were next isolated from WT and p28-deficient mice and compared for their capacity to support SP1 maturation. Compared to the co-culture with WT DCs, the one with p28-deficient DCs gave rise to a significantly reduced number of Qa-2^+^ SP4 cells. More intriguingly, the majority of thymocytes in the culture acquired Qa-2 expression with the addition of exogenous IL-27, regardless of the origin of the thymic DCs (Fig. 4C). These *in vitro* data support an important role of IL-27 in the regulation of SP thymocyte maturation.

### Restored Qa-2^+^ CD4 SP population in p28-deficient mice following intrathymic injection of IL-27

**Figure 5:**
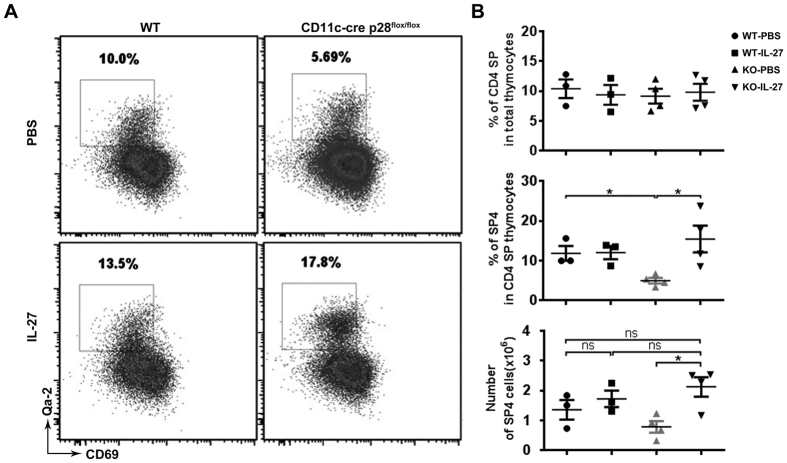
The rescuing effect of intrathymically injected IL-27. CD11c-cre p28^flox/flox^ and WT mice received intrathymic injection of IL-27 or PBS. Two days later, the thymocytes were collected, stained with antibodies for CD4, CD8, CD69, Qa-2 and analyzed by flow cytometry. (**A**) Representative dot plots showing CD69 and Qa-2 expression in CD4 SP thymocytes. (**B**) The percentage of CD4 SP cells in total thymocytes, the percentage of SP4 cells in the CD4 SP population, and the absolute number of SP4 cells are shown. Each symbol represents an individual sample and the horizontal bar denotes the mean. *p < 0.05.

We further tested whether administration of exogenous IL-27 was capable of rescue the maturation defect in CD11c-cre p28^flox/flox^ thymi. Mice received intrathymic injection of recombinant IL-27 and their thymocyte development was evaluated 48 hours after injection. As shown in Fig. 5, treatment with IL-27 resulted in a drastic increase of the Qa-2^+^ SP4 subset in CD11c-cre p28^flox/flox^ mice, raising it to a level comparable to that in WT mice. On the other hand, neither the total CD4 SP thymocyte nor the SP4 subset was significantly altered in WT mice following IL-27 administration. These results support that loss of IL-27 is a direct cause of the defective maturation of CD4 SP thymocytes in p28-deficient mice.

### Qa-2 expression was positively co-related with IFIT1, IFIT3 and IRF7

**Figure 6:**
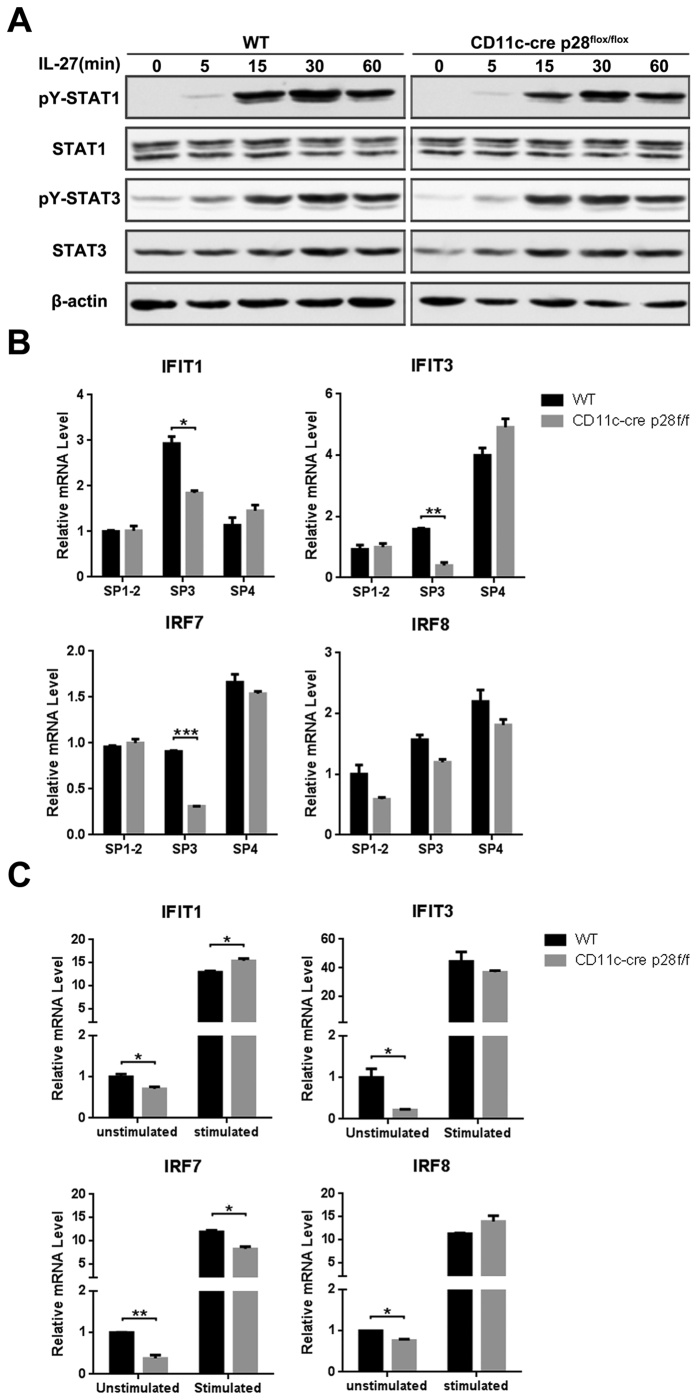
IL27- mediated signal in CD4^+^ SP thymocytes. (**A**) CD4^+^ SP thymocytes were isolated form CD11c-cre p28^flox/flox^ and WT mice, cultured in RPMI-1640 medium with IL-27. Cells were harvested at 0, 5, 15, 30 and 60 min after stimulation, Phosphorylated and total STAT1 and STAT3 were detected by Western blotting. The cropped gels have been run under the same experimental conditions. Representative blots are shown of three independent experiments. β-actin was used as a control. (**B**) SP3 cells from CD11c-cre p28^flox/flox^ mice and WT littermates were stimulated by 2 ng/ml IL-27 for 24 hours, cells were collected and the mRNA expression of IFIT1, IFIT3, IRF7 and IRF8 were evaluated (n ≥ 3). The experiments were repeated for three times and the data are shown as mean ± s.d. (**C**) Total RNA was extracted from FACS-purified CD4^+^ SP thymocyte subsets from CD11c-cre p28^flox/flox^ mice and WT littermates. Quantitative PCR was performed to analyze mRNA expression of IFIT1, IFIT3, IRF7 and IRF8 (n ≥ 3). *p < 0.05; **p < 0.01; ***p < 0.001.

In peripheral T cells, IL-27 conducts signals through interaction with its cognate receptor IL-27RA and gp130 and the subsequent activation of the JAK-STAT pathway^31^,^32^. Both IL-27 receptor subsets IL-27RA and gp130 were abundant in CD4 SP thymocytes(Fig. 1C). We speculate that the JAK-STAT pathway may also be involved in IL-27-induced maturation of CD4 SP cells. Indeed, the tyrosine phosphorylation of STAT1 and STAT3 were found to be significantly elevated when freshly isolated CD4^+^ SP thymocytes were stimulated with IL-27. As expected, p28 deficiency did not altered the response of CD4^+^ SP thymocytes to IL-27 (Fig. 6A).

Our previous transcriptome analyses of CD4 SP thymocytes have demonstrated that there is a sharp increase of multiple gene transcripts at the SP4 stage, many of which are related to the JAK-STAT signaling cascade, such as IFIT1, IFIT3, IRF7 and IRF8^33^ (The microarray data have been deposited in NCBI’s Gene Expression Omnibus. http://www.ncbi.nlm.nih.gov/geo/query/acc.cgi?acc=GSE30083). Notably, IL-27 stimulation induced a drastic increase of their transcription in SP3 cells (Fig. 6B). On the other hand, SP3 cells from p28-deficient mice had a much lower expression of IFIT1, IFIT3, and IRF7 than the wild type counterparts (Fig. 6C). Therefore, these genes may be directly regulated by IL-27 signaling.

## Discussion

The present study revealed a previously undescribed function of IL-27 in the maturation of CD4 SP thymocytes. IL-27 was found to be highly expressed in thymic DCs, while its receptor was mainly detected in CD4 SP thymocytes. When added into cultures, IL-27 was able to drive the phenotypic maturation of newly generated CD4 SP thymocytes into Qa-2^+^ cells. On the contrary, mice with DC-specific deletion of IL-27p28 exhibited a significant reduction of the most mature, Qa-2-expressing CD4 SP thymocytes. Notably, such a defect was rescued following intrathymic injection of exogenous IL-27. These data indicate that thymic DC-derived IL-27 plays an important role in the late stage developmental of thymocytes.

The newly generated SP thymocytes migrate into the thymic medulla, where they undergo phenotypic and functional maturation before being exported to the periphery^9^. Our previous studies have shown that the maturation of CD4 SP cells follows a program composed of multiple intermediate stages^10^. While the progression from SP1 to SP3 seems to be a cell-autonomous process, the transition from SP3 to SP4 apparently requires signals provided by thymic stromal cells^9^,^30^. The molecular nature of the signal, however, remains elusive. The present study provides evidence that IL-27 is involved in the regulation of this transition. An intriguing yet unresolved issue is whether IL-27 drives the full maturation or simply allows the acquisition of Qa-2 expression. Despite the reduced population size, a substantial number of SP4 cells were generated in CD11c-cre p28^flox/flox^ mice. When tested for their functionality, these cells displayed similar proliferative responses and even increased cytokine production in comparison to the wild type cells (data not shown). Therefore, signals other than IL-27 also contribute to the final maturation of CD4 SP thymocytes. Consistent with the observation *in vivo*, the thymic DC isolated from CD11c-cre p28^flox/flox^ mice was able to support the generation of Qa-2^+^ cells from SP1 cells in culture, although less efficient than the wild type DC. As a matter of fact, such a property is not even restricted to dendritic cells. Thymic epithelial cells were also capable of driving the maturation of SP1 cells in culture^9^.

During CD4 SP maturation, three important events were occurred in the medulla, including the differentiation of regulatory T cells, negative selection and thymic emigration. Our results suggest that IL-27 is involved in the late stage thymocytes phenotypic maturation. Therefore, we try to figure out the impact of IL-27 on these critical events. However, using the RIP-mOVA and OT-II model, we observed no change in the deletion of Vα2^+^Vβ5^+^ cells in wild type and p28-deficient mice (supplementary Fig. S1). Meanwhile, we had examined Foxp3 expression in SP thymocytes, no obvious difference was observed for the Treg population in wild type and CD11c-cre p28^flox/flox^ mice (supplementary Fig. S2). Indeed, our ongoing work shows that p28 deficiency does appear to render the mice more susceptible to autoimmune disorders, especially when crossed with Aire-deficient mice (data not shown). Data currently available, however, argues against the possibility of defective negative selection or impaired Treg differentiation. Using the Rag2p-GFP model, we found a decrease in GFP^mid/hi^ cells among CD4^+^ T cells in lymph nodes and spleen in CD11c-cre p28^flox/flox^ mice (supplementary Fig. S3). In adult mice, more that 80% recent thymic emigrants were Qa-2^+^ cells^30^. Therefore, the thymic output is indeed affected by the retarded maturation of Qa-2^+^ SP4 cells in the thymus.

In summary, our study demonstrates that thymic DC-derived IL-27 plays an important role in the phenotype development of CD4 single positive thymocytes. IL-27 and its receptor mediated-STATs signaling is involved in this process. Their contribution to IL-27-induced CD4 SP functional maturation needs further investigation.

## Additional Information

**How to cite this article**: Tang, H. *et al*. Thymic DCs derived IL-27 regulates the final maturation of CD4^+^ SP thymocytes. *Sci. Rep.* **6**, 30448; doi: 10.1038/srep30448 (2016).

## Supplementary Material

Supplementary Information
