# Erratum: Thymic DCs derived IL-27 regulates the final maturation of CD4^+^ SP thymocytes

**DOI:** 10.1038/srep36432

**Published:** 2016-11-11

**Authors:** Hui Tang, Jie Zhang, Xiuyuan Sun, Xiaoping Qian, Yu Zhang, Rong Jin

**Keywords:** Interleukins, CD4-positive T cells

10.1038/srep30448

This Article contains errors. In the PDF version of this Article, the abbreviations for corresponding authors have been reversed

“Correspondence and requests for materials should be addressed to Y.Z. (email: jinrong@bjmu.edu.cn) or R.J. (email: zhangyu007@bjmu.edu.cn)”

should read:

“Correspondence and requests for materials should be addressed to R.J. (email: jinrong@bjmu.edu.cn) or Y.Z. (email: zhangyu007@bjmu.edu.cn)

This is correct in the HTML version of the Article

In the Materials and Methods section under the subheading “Thymic stromal cell isolation” section,

“To purify thymic DCs, cells were stained with fluorescence-labeled antibodies against CD11c, CD45RA, CD8α and CD172α and sorted on a BD FACS Aria II into the following fractions: total DCs (CD11c^+^), pDCs (CD11c^int/hi^CD45RA^+^), resident conventional DCs (CD11c^+^CD8α^+^CD172α^−^), and migratory DCs (CD11c^+^CD8α^−^CD172α^−^)”

should read:

“To purify thymic DCs, cells were stained with fluorescence-labeled antibodies against CD11c, CD45RA, CD8α and CD172α and sorted on a BD FACS Aria II into the following fractions: total DCs (CD11c^+^), pDCs (CD11c^int/hi^CD45RA^+^), resident conventional DCs (CD11c^+^CD8α^+^CD172α^−^), and migratory DCs (CD11c^+^CD8α^−^CD172α^+^)”

In the Results section, the subheading

“Reduction of CD69^−^Qa^−^2^+^ SP4 thymocytes in CD11c-cre p28^flox/flox^ mice”

should read:

“Reduction of CD69^−^Qa−2^+^ SP4 thymocytes in CD11c-cre p28^flox/flox^ mice”

In addition, the figure legend for figure 1(B),

“(B) IL-27 protein expression in pDC, CD8^+^ and CD172^+^ cDC was analyzed by flow cytometry following intracellular staining with anti-IL-27”.

Should read

“(B) IL-27 protein expression in pDC, CD8α^+^ and CD172α^+^ cDC was analyzed by flow cytometry following intracellular staining with anti-IL-27”.

Figure 6B and 6C were inverted. The correct Figure 6 appears below as Figure 1.

**Figure 1:**
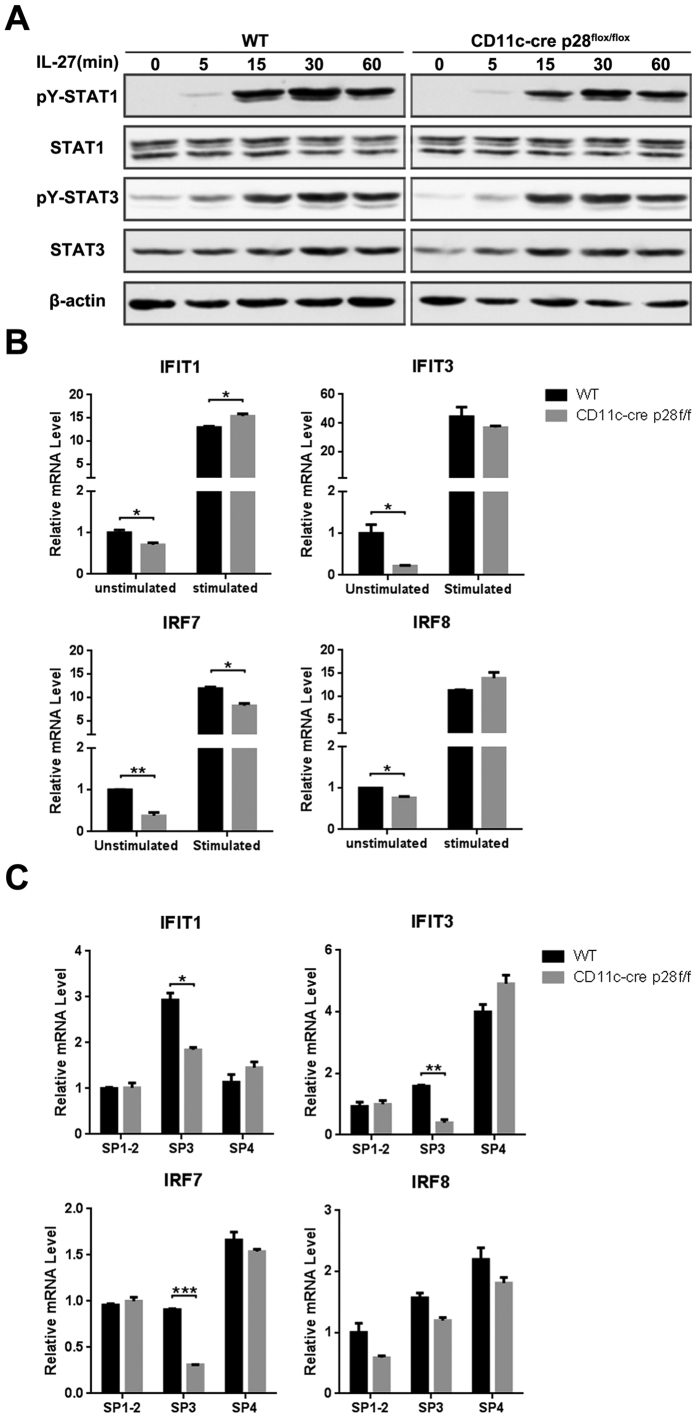
Figure 1.

