# Supplementary material for: Thymic DCs derived IL-27 regulates the final maturation of CD4+ SP thymocytes
Source: Sci Rep. 2016 Jul 29;6:30448. doi: 10.1038/srep30448 (PMC5387111; doi:10.1038/srep30448)
Supplement: Supplementary Information [file srep30448-s1.doc]

Title

Thymic DCs derived IL-27 regulates the final maturation of CD4^+^ SP thymocytes

Author Information

Hui Tang^1^, Jie Zhang, Xiuyuan Sun, Xiaoping Qian, Yu Zhang*, Rong Jin*

Key Laboratory of Medical Immunology, Department of Immunology, Ministry of Health, School of Basic Medical Sciences, Peking University Health Science Center, 38 Xue Yuan Road, Beijing, China

*Correspondence: Drs. Rong Jin or Yu Zhang, Department of Immunology, Peking University, 38 Xue Yuan Road, Beijing 100191, China.

E-mail: jinrong@bjmu.edu.cn or [zhangyu007@bjmu.edu.cn](mailto:zhangyu007@bjmu.edu.cn)

Supplementary Table 1. Nucleotide sequence of real-time qPCR primers

| Gene | Sense(5’→3’) | Anti-Sence(5’→3’) |
| --- | --- | --- |
| p28 | CTCTGCTTCCTCGCTACCAC | GGGGCAGCTTCTTTTCTTCT |
| Ebi3 | TCCTAGCCTTTGTGGCTGAG | GAGAAGATGTCCGGGAAGG |
| IL-27Ra | TGTGAAACTTCTGGCAAACG | CGGGACCTCTTCTTGAGTTG |
| gp130 | CTGAGGGACCGGTGGTGT | GAAACAAGTTCGCTGCCTCT |
| IFIT1 | CAAGGCAGGTTTCTGAGGAG | AGCTTCCATGTGAAGTGACATCT |
| IFIT3 | TTCCCAGCAGCACAGAAAC | TGCACACCCTGTCTTCCATA |
| IRF7 | ACAGCACAGGGCGTTTTATC | AGCATTGCTGAGGCTCACTT |
| IRF8 | GATCGAACAGATCGACAGCA | AGAGCACAGCGTAACCTCGT |
| GAPDH | CGGCCGCATCTTCTTGTGCA | GCAAATGGCAGCCCTGGTGAC |


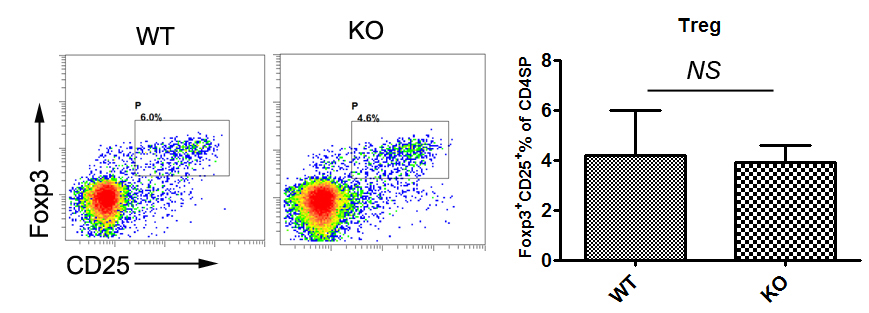


Supplementary Fig 1. The development of regulatory T cells in CD11c-cre p28^-flox/flox^ mice. Thymocytes were collected from 6 weeks of age mice, and stained with anti-CD4, anti-CD8, anti-CD25 and anti-Foxp3. CD4^+^CD8^-^ thymocytes were gated for the expression of CD25 and Foxp3 (left panal). The percentage of regulatory T cells in the CD4 single positive thymocytes was shown in the right panal. The data are shown as mean±s.d. (N=3). NS, no significance.


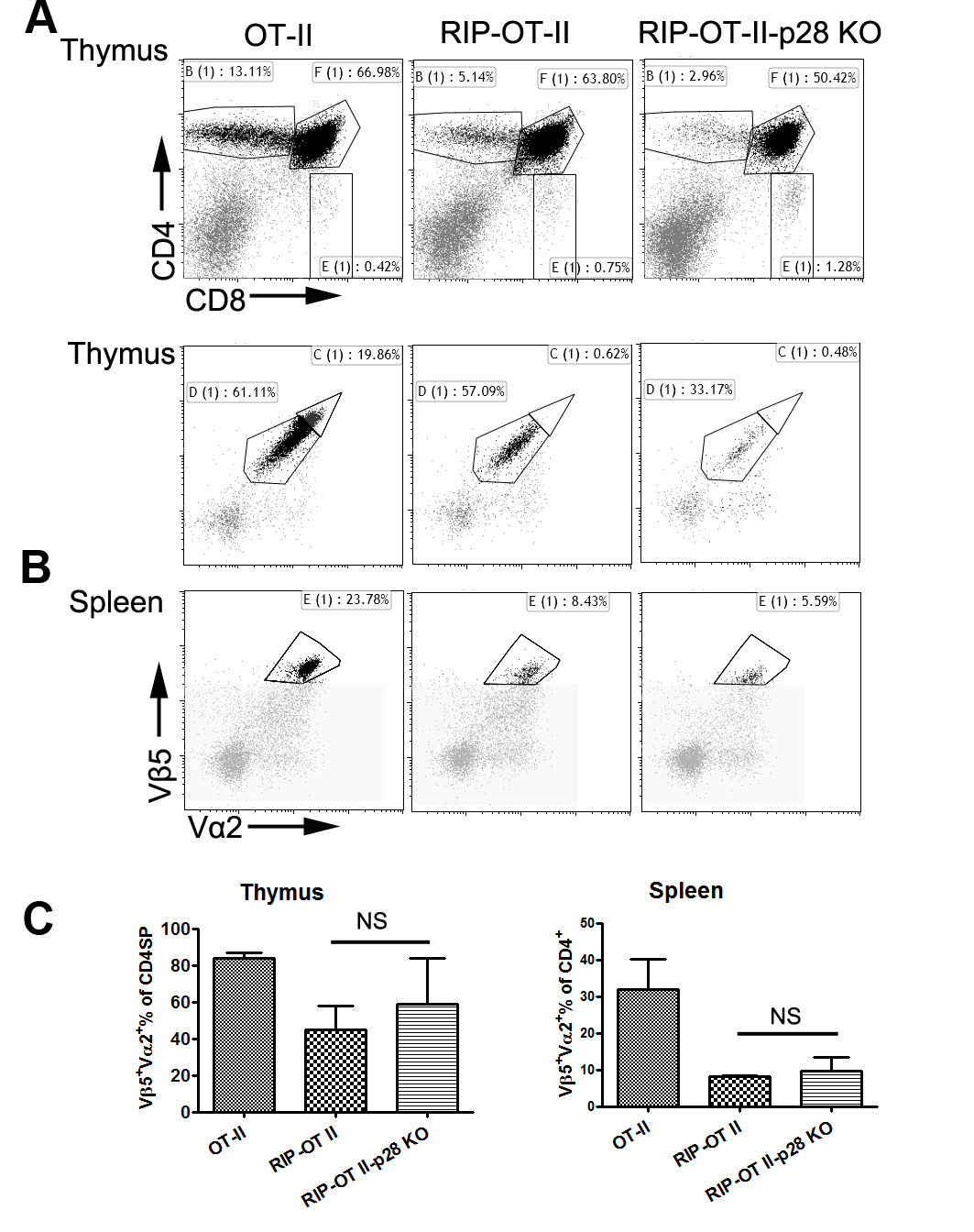


Supplementary Fig 2. The role IL-27 in the negative selection. OT-II TCR tg and OT-II-RIP-mOVA double-tg mice were crossed with CD11c-cre p28^-flox/flox^ mice. Thymocytes and splenocytes from 5- to 6-week-old mice were collected and stained with anti-CD4, anti-CD8, and the anticlonotypic combination of anti-Vα2 TCR and anti-Vβ5 TCR mAbs. (*A*) The upper panel dot plots showed the CD4 and CD8 expression in total thymocytes. The lower panel dot plots showed the Vα2 and Vβ5 expression in CD4SP thymocytes. (*B*) Representative dot plots showing Vα2 and Vβ5 expression in CD4^+^ T cells in the spleen. (*C*) The percentage of Vα2^+^Vβ5^+^ cells in the CD4 SP thymocytes, and the percentage of Vα2^+^Vβ5^+^ cells in the CD4^+^ T cells in the spleen. (n=3). The data are shown as mean±s.d. NS, no significance.


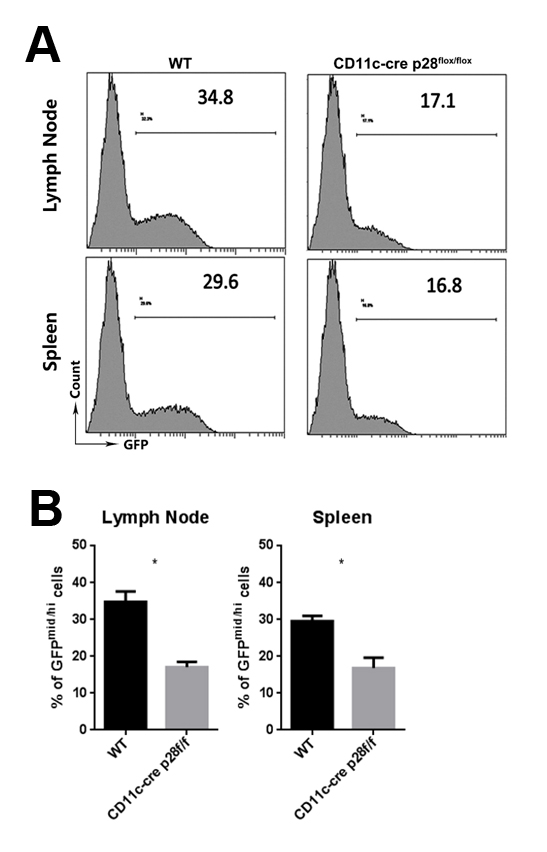


Supplementary Fig 3. The thymic output in CD11c-cre p28^-flox/flox^ RAG2p-GFP mice (N=5). Spleen and lymph nodes from 5- to 6-week-old mice were collected and analyzed the GFP expreesion in CD4^+^ T cells. (A) Representative histogram showing GFP expression in CD4^+^ T cells in the Spleen and lymph nodes. (*B*) The percentage of GFP^+^ cells in in CD4^+^ T cells in the Spleen and lymph nodes. The data are shown as mean±s.d. **p*<0.05.
